# Supplementary material for: Computerised cognitive training in Parkinson’s disease: a protocol for a systematic review and updated meta-analysis
Source: BMJ Open. 2020 Nov 24;10(11):e040656. doi: 10.1136/bmjopen-2020-040656 (PMC7689075; doi:10.1136/bmjopen-2020-040656)
Supplement: Author's manuscript [file bmjopen-2020-040656.draft_revisions.pdf]

BMJ Open

BMJ Open is committed to open peer review. As part of this commitment we make the peer review history of every article we publish publicly available.

When an article is published we post the peer reviewers' comments and the authors' responses online. We also post the versions of the paper that were used during peer review. These are the versions that the peer review comments apply to.

The versions of the paper that follow are the versions that were submitted during the peer review process. They are not the versions of record or the final published versions. They should not be cited or distributed as the published version of this manuscript.

BMJ Open is an open access journal and the full, final, typeset and author-corrected version of record of the manuscript is available on our site with no access controls, subscription charges or pay-per-view fees (http://bmjopen.bmj.com).

If you have any questions on BMJ Open's open peer review process please email info.bmjopen@bmj.com

BMJ Open

Computerized Cognitive Training in Parkinson's Disease: A Protocol for a Systematic Review and Updated Meta-Analysis

Journal:	*BMJ Open*
Manuscript ID	bmjopen-2020-040656
Article Type:	Protocol
Date Submitted by the Author:	19-May-2020
Complete List of Authors:	Malmberg Gavelin, Hanna; University of Melbourne Academic Unit for Psychiatry of Old Age; Umea University, Department of Psychology<br>Domellöf, Magdalena; Umea University, Department of Psychology<br>Leung, Isabella; The University of Sydney, Healthy Brain Ageing Program, Brain and Mind Centre; The University of Sydney, Central Clinical School, Faculty of Medicine and Health, Charles Perkins Centre<br>Stigsdotter Neely, Anna; Karlstad University, Department of Social and Psychological Studies<br>Finke, Carsten; Charite Universitatsmedizin Berlin, Department of Neurology; Humboldt-Universitat zu Berlin, Berlin School of Mind and Brain<br>Lampit, Amit; University of Melbourne Academic Unit for Psychiatry of Old Age; Charite Universitatsmedizin Berlin, Department of Neurology
Keywords:	Parkinson-s disease < NEUROLOGY, REHABILITATION MEDICINE, GERIATRIC MEDICINE

SCHOLARONE™
Manuscripts

I, the Submitting Author has the right to grant and does grant on behalf of all authors of the Work (as defined in the below author licence), an exclusive licence and/or a non-exclusive licence for contributions from authors who are: i) UK Crown employees; ii) where BMJ has agreed a CC-BY licence shall apply, and/or iii) in accordance with the terms applicable for US Federal Government officers or employees acting as part of their official duties; on a worldwide, perpetual, irrevocable, royalty-free basis to BMJ Publishing Group Ltd ("BMJ") its licensees and where the relevant Journal is co-owned by BMJ to the co-owners of the Journal, to publish the Work in this journal and any other BMJ products and to exploit all rights, as set out in our licence.

The Submitting Author accepts and understands that any supply made under these terms is made by BMJ to the Submitting Author unless you are acting as an employee on behalf of your employer or a postgraduate student of an affiliated institution which is paying any applicable article publishing charge ("APC") for Open Access articles. Where the Submitting Author wishes to make the Work available on an Open Access basis (and intends to pay the relevant APC), the terms of reuse of such Open Access shall be governed by a Creative Commons licence – details of these licences and which Creative Commons licence will apply to this Work are set out in our licence referred to above.

Other than as permitted in any relevant BMJ Author's Self Archiving Policies, I confirm this Work has not been accepted for publication elsewhere, is not being considered for publication elsewhere and does not duplicate material already published. I confirm all authors consent to publication of this Work and authorise the granting of this licence.

For peer review only

Computerized Cognitive Training in Parkinson's Disease: A Protocol for a Systematic Review and Updated Meta-Analysis

Hanna Malmberg Gavelin[1, 2], Magdalena Domellöf[2], Isabella Leung[3, 4], Anna Stigsdotter Neely[5], Carsten Finke[6, 7], Amit Lampit[1, 6, 7]*

[1] Academic Unit for Psychiatry of Old Age, University of Melbourne, Parkville, Australia

[2] Department of Psychology, Umeå University, Umeå, Sweden

[3] Healthy Brain Ageing Program, Brain and Mind Centre, University of Sydney, Camperdown, Australia

[4] Central Clinical School, Faculty of Medicine and Health, Charles Perkins Centre, University of Sydney, Camperdown, Australia

[5] Department of Social and Psychological Studies, Karlstad University, Karlstad, Sweden

[6] Department of Neurology, Charité – Universitätsmedizin Berlin, Berlin Germany

[7] Berlin School of Mind and Brain, Humboldt-Universität zu Berlin, Berlin, Germany

* Correspondence concerning this article should be addressed to Amit Lampit, 151 Barry Street, Carlton South VIC 3053, Australia, amit.lampit@unimelb.edu.au

Keywords: Parkinson's disease, cognitive training, systematic review, meta-analysis

Word count: 1988

For peer review only

Abstract

Introduction. Cognitive impairment is recognized as an important non-motor symptom in Parkinson's disease (PD) and there is a need for evidence-based non-pharmacological interventions that may prevent or slow cognitive decline in this patient group. One such intervention is computerized cognitive training (CCT), which has shown efficacious for cognition across older adult populations. This systematic review aims to investigate the efficacy of CCT across cognitive, psychosocial and functional domains for people with PD, and examine study and intervention design factors that could moderate CCT effects on cognition.

Methods and analysis. Randomized controlled trials investigating the effects of CCT in patients with PD without dementia, on cognitive, psychosocial or functional outcomes will be included. The primary outcome is overall cognitive function. Secondary outcomes are domain-specific cognitive function, psychosocial functioning and functional abilities. We will systematically search MEDLINE, Embase and PsycINFO to identify relevant literature. Risk of bias will be assessed using the revised Cochrane Risk of Bias tool. Effect sizes will be calculated as standardized mean difference of baseline to post-intervention change (Hedges' *g*) with 95% confidence interval for each eligible outcome measure. Pooling of outcomes across studies will be conducted using random-effects models, accounting for dependency structure of effect sizes within studies. Heterogeneity will be assessed using tau^2 and I^2 statistic. Potential moderators, based on key study and intervention design factors, will be investigated using mixed-effects meta-regression models.

Ethics and dissemination. No ethical approval is required. The findings will be disseminated in a peer-reviewed scientific journal.

For peer review only - http://bmjopen.bmj.com/site/about/guidelines.xhtml

ARTICLE SUMMARY

Strengths and limitations of this study

- Specification of the eligibility criteria for randomized controlled trials of narrowly-defined computerized cognitive training delineates the effects of this intervention from other cognitive intervention approaches in Parkinson's disease.
- Inclusion of a variety of cognitive, psychosocial and functional outcome measures will improve statistical power to inform efficacy across and within domains.
- Accounting for dependency of effect sizes within studies will reduce overestimation of within-study variance and thus underestimation of between-study heterogeneity, especially since individual studies are expected to be underpowered.
- Multiple methods for investigating heterogeneity can inform intervention and study design, but contingent on the number and size of available studies.
- Analyses are limited to group- rather than individual participant data.

For peer review only

INTRODUCTION

Cognitive decline is one of the most common non-motor symptoms in Parkinson's disease (PD).[1] Approximately 20% of people with PD already have mild cognitive impairment (MCI) at diagnosis, with over 40% conversion to dementia 10 years after PD diagnosis, substantially exceeding age-standardized risk.[1] Considering the negative influence of cognitive impairment on quality of life for patients as well as caregivers and the current lack of effective pharmacological treatments,[1] developing interventions that could maintain cognitive function and delay cognitive and functional decline is a critical area for prevention and treatment research in the field.[2]

Cognitive training is a non-pharmacological intervention that has shown efficacious for cognition in older adults across the spectrum from cognitively healthy to dementia.[3] Specifically, computerized cognitive training (CCT) has received widespread attention in recent years as a safe and scalable intervention that can incorporate important intervention design features such as adaptivity of training difficulty and continuous motivational feedback on training performance.[4] In addition to efficacy for cognition, individual trials have reported potential benefits on other symptoms such as mood and freezing of gait.[4]

To date, several systematic reviews and meta-analyses have investigated the efficacy of cognitive training on cognitive function in PD. Leung and colleagues[5] identified seven randomized controlled trials (RCTs) and concluded that cognitive training showed modest efficacy for overall cognition in PD, with larger effect sizes observed within individual cognitive domains.[5] Similar results were reported by Lawrence and colleagues,[6] who combined 11 randomized and non-randomized cognitive training trials. More recently, a Cochrane review identified seven RCTs investigating the effects of cognitive training in PD patients with MCI or dementia, reporting imprecise and uncertain evidence for efficacy on

For peer review only - http://bmjopen.bmj.com/site/about/guidelines.xhtml

global cognition.[7] Approaches for estimating effect sizes across studies varied across reviews, and none conducted investigations of heterogeneity.

Taken together, previous reviews have shown mixed and inconclusive results and the efficacy of cognitive training in general, and CCT in particular, in people with PD remains uncertain. Furthermore, given the limited number of studies in previous reviews as well as clinical and methodological heterogeneity, the effects of CCT across different cognitive, psychosocial and functional domains as well as design factors that may associated with such effects are still unclear.[4]

Objectives

The aim of this review is to evaluate the efficacy of CCT on cognitive, psychosocial and functional outcomes in persons with PD. Specifically, we aim to:

1. Investigate the efficacy of CCT on cognitive, psychosocial and daily function in PD, in comparison to active or passive control.
2. Examine study and intervention design factors that could moderate CCT effects on cognitive function across studies.
3. Evaluate the strength and quality of the evidence for CCT in PD.
4. Suggest recommendations for future research and practice in the field.

METHODS AND ANALYSIS

This protocol adheres to the Preferred Reporting Items for Systematic Review and Meta-analysis Protocols (PRISMA-P) guidelines.[8] The PRISMA-P checklist can be found in the online supplementary information. This review updates and further specifies our previous systematic review on cognitive training in PD.[5]

For peer review only

Eligibility criteria

Consistent with our previous systematic reviews of CCT,[9-11] we will include studies that meet the following criteria:

Types of studies

RCTs studying the effects of CCT on one or more cognitive, psychosocial or functional outcome in patients with PD. Eligible studies will provide neuropsychological testing at baseline and post CCT intervention. Randomised crossover trials will be included but only the first treatment phase will be considered and used for analysis. Non-randomised trials will be excluded. Unpublished RCTs or those published as conference abstracts, theses or monographs will be eligible if data needed for analysis and appraisal can be obtained from the authors.

Types of participants

Patients with PD (any age and aetiology), either cognitively healthy, with subjective cognitive decline or MCI. Studies including only or mainly people with dementia will be excluded. Studies reporting the results from a population with mixed populations (e.g., MCI and dementia) will be eligible if the results for the eligible population are reported or can be obtained separately or if the eligible population (e.g., PD with MCI) constitutes ≥50% of the sample.

Types of interventions

Minimum of 4 h of practice on standardized computerized tasks or video games with clear cognitive rationale, administered on personal computers, mobile devices or gaming consoles. Studies combining CCT with other non-pharmacological interventions (e.g., physical

For peer review only

exercise, brain stimulation) or with pharmacological interventions will be eligible as long as the CCT condition is the only key difference between the two groups (i.e., study design allows to delineate the effect of CCT from the composite intervention). Studies will not be included if: (1) more than 50% of total intervention time was not CCT; (2) the intervention does not involve interaction with a computer (e.g., passive viewing or recording of responses by an experimenter); (3) the CCT intervention is based on lab-specific rather than off-the-shelf hardware, which makes it less likely to be relevant to clinical practice.

Types of comparators

Eligible control conditions include wait-list, no-contact and active (e.g., sham CCT, recreational activities) control groups. Combined interventions (e.g., pharmacological, physical exercise) will be eligible if provided similarly to both groups. All eligible controls in multi-arm studies will be included.

Types of outcomes

Eligible outcomes are change in performance from baseline to post-intervention in non-trained measures of cognition (global or domain-specific), assessed through standardised neuropsychological tests or close equivalents (e.g., a computer-based version of a common neuropsychological test). Additional outcomes include quality of life (standardised psychological wellbeing and quality of life questionnaires), mood (e.g., clinical depression rating scales), subjective cognition and daily function (patient or informant reported activities of daily living questionnaires or standardised measures, e.g., timed IADL). Outcomes will be excluded if they were used as (or closely resemble) training tasks or exploratory in nature (i.e., do not resemble common neuropsychological tests). In studies reporting more than one outcome measure per category, all eligible outcome measures will be included. The primary

For peer review only

outcome will be overall cognitive performance. [5 9-11] Secondary outcomes are domain-specific cognitive performance, classified according to the CHC-M framework,[12] subjective cognition, psychosocial functioning and daily function.

Search strategy

We will search MEDLINE, EMBASE and PsycINFO through the OVID interface for eligible articles. As this is an update of our previous systematic review,[5] the search will be limited to entries from 1 January 2015. No restrictions on language or type of publication will be applied. The electronic search will be complemented by hand-searching the references of included articles and previous reviews as well as clinical trial registries. The full search strategy is shown in Table 1.

Table 1

Search strategy

# 1	((cognit* or attention or neurocognit* or neuropsycholog* or memory or mental or reasoning or executive) adj2 (interven* or training* or rehabilitat* or remediat* or stimulat* or activit* or enhanc* or exercis* or retrain*)).mp.
# 2	((brain) adj2 (training* or rehabilitat* or remediat* or retrain*)).mp.
# 3	(speed adj3 training).mp
# 4	(video gam* or videogam* or wii or computer gam* or virtual reality).mp.
# 5	1 or 2 or 3 or 4
# 6	parkinson$.mp
# 7	exp Parkinson's disease/
# 8	exp Parkinsonism/
# 9	6 or 7 or 8
# 10	5 and 9
# 11	limit 10 to yr="2015 -Current"

Study selection

Literature search results will be uploaded to a single Covidence library. Duplicates will be removed and articles identified from other sources will be added. Initial screening for eligibility based on titles and abstracts will be conducted by two independent reviewers. Full-

For peer review only - http://bmjopen.bmj.com/site/about/guidelines.xhtml

text screening of potentially relevant articles will be conducted by two independent reviewers and disagreements resolved by consensus or by involvement of a third reviewer.

Data extraction

Data will be extracted to a piloted Excel spreadsheet by one reviewer and a senior reviewer will check the data. Any disagreements will be resolved by consensus or by involvement of a third reviewer if necessary. If any additional information is needed, we will contact the corresponding authors of the studies. The following data items will be extracted:

- Study information: first author, year of publication, study location
- Population: mean age, percent male, mean MMSE score (or equivalent), mean Unified Parkinson's Disease Rating score (or equivalent), disease stage (Hoehn & Yahr Scale or equivalent), disease duration, medication use, cognitive status (normal, subjective cognitive complaints or MCI)
- Intervention: type of CCT, program used, training content, delivery format (supervised or unsupervised), total training duration (hours), session frequency (sessions/week), session length (minutes), total number of sessions, intervention duration (weeks), adjacent treatments
- Comparator: type of control, control group activity
- Outcome: name of test, summary data for each group (e.g., mean, standard deviation, sample size) at baseline and post intervention, cognitive domain

Intention-to-treat data will be preferred if reported. Data will be extracted as means and standard deviation for each time point if reported. If such information is not available, data in other formats (e.g., mean change and standard deviation) will be used if the article provides

For peer review only

sufficient information to reliably calculate standardised mean difference. If these data are unavailable, authors will be contacted to obtain missing data.

Risk of bias assessment

Risk of bias in individual RCTs will be assessed using the revised Cochrane Risk of Bias tool (RoB 2).[13] Low, high or some concerns risk of bias will be determined for each of the following domains:

1. Bias arising from the randomization process
2. Bias due to deviations from intended interventions
3. Bias due to missing outcome data
4. Bias in measurement of the outcome
5. Bias in selection of the reported result
6. Overall bias

Studies with "some concerns" or "high" risk of bias in domains 3 or 4 will be considered as having some concerns or high risk of bias, respectively. Two independent reviewers will assess the risk of bias and disagreements will be resolved by consensus or consulting a third reviewer if necessary.

Data synthesis

Analyses will be conducted using the packages metafor, metaSEM, robumeta and clubSandwich for R. Between-group differences in change from baseline to post-intervention will be converted to standardized mean differences and calculated as Hedges' *g* with 95% confidence interval for each eligible outcome measure. Pooling of outcomes across studies will be conducted using random-effects models, accounting for dependency structure of effect sizes within studies.[14 15] Sensitivity analyses for the primary outcome will be conducted by

For peer review only

comparing results from multilevel and robust variance estimation models. Analyses of secondary outcomes will be contingent on the availability of at least three studies for analysis.

Heterogeneity across studies will be quantified using tau^2 and expressed as a proportion of overall observed variance using the I^2 statistic.[16 17] Prediction intervals will be calculated to assess the dispersion of effects across settings.[18] Provided sufficient statistical power for investigations of heterogeneity,[19] potential moderators will be investigated using mixed-effects meta-regression models. The following moderators will be tested, if warranted: training content and type; control content and type; population (clinical or cognitive status); delivery format; training dose and frequency. If warranted, potential interactions across moderators will be tested on an exploratory basis using multivariate meta-regression or network meta-analysis.

Meta-bias(es)

Small-study effect will be assessed by visually inspecting funnel plots of effect size vs standard error. [20] If at least 10 studies are available, small study effect will be formally tested using a multivariate analogue of the Egger's test,[21] i.e., a meta-regression using standard error as covariate. Subgroup analysis of the primary outcome will be conducted based on overall RoB 2 scores.

Confidence in cumulative evidence

The strength of the evidence will be assessed and summarized qualitatively based on risk of bias for individual studies, precision of the effect estimates, heterogeneity across studies (including prediction intervals) and evidence for small study effects, with additional sensitivity analyses conducted if warranted.

For peer review only

Patient and public involvement

Patients and/or the public will not be involved in this study.

Ethics and dissemination

No formal ethical assessment or informed consent is required for this study. The findings of the study will be summarised in a manuscript which will be submitted for publication in a peer-reviewed scientific journal.

For peer review only

Author Contributions. Guarantor: AL. Design and conceptualisation: HMG and AL. Data collection: HMG and MD. Risk of bias assessment: HMG and MD. Data analysis and interpretation: HMG, MD, IL, ASN, CF and AL. Drafting and revising the manuscript: HMG, MD, IL, ASN, CF and AL.

Funding. This work was supported by a CR Roper Fellowship from the University of Melbourne provided to AL (2020-1), and by the Swedish Research Council (2017-02371) as well as the Swedish Research Council for Health, Working-Life and Welfare (2014-01654) awarded to ASN.

Competing interests. None declared.

References

1. Aarsland D, Creese B, Politis M, et al. Cognitive decline in Parkinson disease. *Nat Rev Neurol* 2017;13(4):217-31. doi: 10.1038/nrneurol.2017.27.

2. Goldman JG, Vernaleo BA, Camicioli R, et al. Cognitive impairment in Parkinson's disease: a report from a multidisciplinary symposium on unmet needs and future directions to maintain cognitive health. *NPJ Parkinsons Dis* 2018;4:19. doi: 10.1038/s41531-018-0055-3.

3. Gavelin HM, Lampit A, Hallock H, et al. Cognition-Oriented Treatments for Older Adults: a Systematic Overview of Systematic Reviews. *Neuropsychol Rev* 2020 doi: 10.1007/s11065-020-09434-8.

4. Walton CC, Naismith SL, Lampit A, et al. Cognitive Training in Parkinson's Disease. *Neurorehabil Neural Repair* 2017;31(3):207-16. doi: 10.1177/1545968316680489.

5. Leung IH, Walton CC, Hallock H, et al. Cognitive training in Parkinson disease: A systematic review and meta-analysis. *Neurology* 2015;85(21):1843-51. doi: 10.1212/WNL.0000000000002145.

6. Lawrence BJ, Gasson N, Bucks RS, et al. Cognitive training and noninvasive brain stimulation for cognition in Parkinson's disease: a meta-analysis. *Neurorehabil Neural Repair* 2017;31(7):597-608. doi: 10.1177/1545968317712468.

7. Orgeta V, McDonald KR, Poliakoff E, et al. Cognitive training interventions for dementia and mild cognitive impairment in Parkinson's disease. *Cochrane Database Syst Rev* 2020;2:CD011961. doi: 10.1002/14651858.CD011961.pub2.

8. Moher D, Shamseer L, Clarke M, et al. Preferred reporting items for systematic review and meta-analysis protocols (PRISMA-P) 2015 statement. *Systematic Reviews* 2015;4:1. doi: 10.1186/2046-4053-4-1.

For peer review only

9. Lampit A, Hallock H, Valenzuela M. Computerized cognitive training in cognitively healthy older adults: a systematic review and meta-analysis of effect modifiers. *PLoS Med* 2014;11(11):e1001756. doi: 10.1371/journal.pmed.1001756.

10. Hill NT, Mowszowski L, Naismith SL, et al. Computerized Cognitive Training in Older Adults With Mild Cognitive Impairment or Dementia: A Systematic Review and Meta-Analysis. *Am J Psychiatry* 2017;174(4):329-40. doi: 10.1176/appi.ajp.2016.16030360.

11. Lampit A, Heine J, Finke C, et al. Computerized Cognitive Training in Multiple Sclerosis: A Systematic Review and Meta-analysis. *Neurorehabil Neural Repair* 2019;33(9):695-706. doi: 10.1177/1545968319860490.

12. Webb SL, Loh V, Lampit A, et al. Meta-Analysis of the Effects of Computerized Cognitive Training on Executive Functions: a Cross-Disciplinary Taxonomy for Classifying Outcome Cognitive Factors. *Neuropsychol Rev* 2018;28(2):232-50. doi: 10.1007/s11065-018-9374-8.

13. Sterne JAC, Savovic J, Page MJ, et al. RoB 2: a revised tool for assessing risk of bias in randomised trials. *BMJ* 2019;366:l4898. doi: 10.1136/bmj.l4898.

14. Cheung MW. A Guide to Conducting a Meta-Analysis with Non-Independent Effect Sizes. *Neuropsychol Rev* 2019;29(4):387-96. doi: 10.1007/s11065-019-09415-6.

15. Hedges LV, Tipton E, Johnson MC. Robust variance estimation in meta-regression with dependent effect size estimates. *Res Synth Methods* 2010;1(1):39-65. doi: 10.1002/jrsm.5.

16. Borenstein M, Higgins JP, Hedges LV, et al. Basics of meta-analysis: I(2) is not an absolute measure of heterogeneity. *Res Synth Methods* 2017;8(1):5-18. doi: 10.1002/jrsm.1230.

For peer review only

17. Higgins JP, Thompson SG. Quantifying heterogeneity in a meta-analysis. *Stat Med* 2002;21(11):1539-58. doi: 10.1002/sim.1186.

18. Riley RD, Higgins JP, Deeks JJ. Interpretation of random effects meta-analyses. *BMJ* 2011;342:d549. doi: 10.1136/bmj.d549.

19. Hedges LV, Pigott TD. The power of statistical tests for moderators in meta-analysis. *Psychol Methods* 2004;9(4):426-45. doi: 10.1037/1082-989X.9.4.426.

20. Sterne JA, Sutton AJ, Ioannidis JP, et al. Recommendations for examining and interpreting funnel plot asymmetry in meta-analyses of randomised controlled trials. *BMJ* 2011;343:d4002. doi: 10.1136/bmj.d4002.

21. Egger M, Davey Smith G, Schneider M, et al. Bias in meta-analysis detected by a simple, graphical test. *BMJ* 1997;315(7109):629-34.

For peer review only

Reporting checklist for protocol of a systematic review.

Based on the PRISMA-P guidelines.

Instructions to authors

Complete this checklist by entering the page numbers from your manuscript where readers will find each of the items listed below.

Your article may not currently address all the items on the checklist. Please modify your text to include the missing information. If you are certain that an item does not apply, please write "n/a" and provide a short explanation.

Upload your completed checklist as an extra file when you submit to a journal.

In your methods section, say that you used the PRISMA-Preporting guidelines, and cite them as:

Moher D, Shamseer L, Clarke M, Ghersi D, Liberati A, Petticrew M, Shekelle P, Stewart LA. Preferred Reporting Items for Systematic Review and Meta-Analysis Protocols (PRISMA-P) 2015 statement. Syst Rev. 2015;4(1):1.

		Reporting Item	Page Number
**Title**			
Identification	#1a	Identify the report as a protocol of a systematic review	1
Update	#1b	If the protocol is for an update of a previous systematic review, identify as such	1

For peer review only

**Registration**			
	#2	If registered, provide the name of the registry (such as PROSPERO) and registration number	Registered with PROSPERO on May 14th 2020, currently pending approval by the PROSPERO editorial team
**Authors**			
Contact	#3a	Provide name, institutional affiliation, e-mail address of all protocol authors; provide physical mailing address of corresponding author	1
Contribution	#3b	Describe contributions of protocol authors and identify the guarantor of the review	12
**Amendments**			
	#4	If the protocol represents an amendment of a previously completed or published protocol, identify as such and list changes; otherwise, state plan for documenting important protocol amendments	Amendments will be recorded in PROSPERO (currently pending approval)
**Support**			
Sources	#5a	Indicate sources of financial or other support for the review	12

For peer review only

Sponsor	#5b	Provide name for the review funder and / or sponsor	12
Role of sponsor or funder	#5c	Describe roles of funder(s), sponsor(s), and / or institution(s), if any, in developing the protocol	n/a
**Introduction**			
Rationale	#6	Describe the rationale for the review in the context of what is already known	4-5
Objectives	#7	Provide an explicit statement of the question(s) the review will address with reference to participants, interventions, comparators, and outcomes (PICO)	5
**Methods**			
Eligibility criteria	#8	Specify the study characteristics (such as PICO, study design, setting, time frame) and report characteristics (such as years considered, language, publication status) to be used as criteria for eligibility for the review	6-8
Information sources	#9	Describe all intended information sources (such as electronic databases, contact with study authors, trial registers or other grey	8

For peer review only

		literature sources) with planned dates of coverage	
Search strategy	#10	Present draft of search strategy to be used for at least one electronic database, including planned limits, such that it could be repeated	8
Study records - data management	#11a	Describe the mechanism(s) that will be used to manage records and data throughout the review	9
Study records - selection process	#11b	State the process that will be used for selecting studies (such as two independent reviewers) through each phase of the review (that is, screening, eligibility and inclusion in meta-analysis)	9
Study records - data collection process	#11c	Describe planned method of extracting data from reports (such as piloting forms, done independently, in duplicate), any processes for obtaining and confirming data from investigators	9-10
Data items	#12	List and define all variables for which data will be sought (such as PICO items, funding sources), any pre-planned data assumptions and simplifications	9-10

For peer review only

Outcomes and prioritization	#13	List and define all outcomes for which data will be sought, including prioritization of main and additional outcomes, with rationale	7-8
Risk of bias in individual studies	#14	Describe anticipated methods for assessing risk of bias of individual studies, including whether this will be done at the outcome or study level, or both; state how this information will be used in data synthesis	10
Data synthesis	#15a	Describe criteria under which study data will be quantitatively synthesised	10-11
Data synthesis	#15b	If data are appropriate for quantitative synthesis, describe planned summary measures, methods of handling data and methods of combining data from studies, including any planned exploration of consistency (such as I2, Kendall's τ)	10-11
Data synthesis	#15c	Describe any proposed additional analyses (such as sensitivity or subgroup analyses, meta-regression)	10-11
Data synthesis	#15d	If quantitative synthesis is not appropriate, describe the type of summary planned	n/a

For peer review only

Meta-bias(es)	#16	Specify any planned assessment of meta-bias(es) (such as publication bias across studies, selective reporting within studies)	11
Confidence in cumulative evidence	#17	Describe how the strength of the body of evidence will be assessed (such as GRADE)	11

The PRISMA-P checklist is distributed under the terms of the Creative Commons Attribution License CC-BY 4.0. This checklist was completed on 15. May 2020 using https://www.goodreports.org/, a tool made by the EQUATOR Network in collaboration with Penelope.ai

For peer review only

BMJ Open

Computerized Cognitive Training in Parkinson's Disease: A Protocol for a Systematic Review and Updated Meta-Analysis

Journal:	*BMJ Open*
Manuscript ID	bmjopen-2020-040656.R1
Article Type:	Protocol
Date Submitted by the Author:	11-Aug-2020
Complete List of Authors:	Malmberg Gavelin, Hanna; University of Melbourne Academic Unit for Psychiatry of Old Age; Umea University, Department of Psychology<br>Domellöf, Magdalena; Umea University, Department of Psychology<br>Leung, Isabella; The University of Sydney, Healthy Brain Ageing Program, Brain and Mind Centre; The University of Sydney, Central Clinical School, Faculty of Medicine and Health, Charles Perkins Centre<br>Stigsdotter Neely, Anna; Karlstad University, Department of Social and Psychological Studies<br>Finke, Carsten; Charite Universitatsmedizin Berlin, Department of Neurology; Humboldt-Universitat zu Berlin, Berlin School of Mind and Brain<br>Lampit, Amit; University of Melbourne Academic Unit for Psychiatry of Old Age; Charite Universitatsmedizin Berlin, Department of Neurology
**Primary Subject Heading**:	Neurology
Secondary Subject Heading:	Rehabilitation medicine
Keywords:	Parkinson-s disease < NEUROLOGY, REHABILITATION MEDICINE, GERIATRIC MEDICINE

SCHOLARONE™
Manuscripts

I, the Submitting Author has the right to grant and does grant on behalf of all authors of the Work (as defined in the below author licence), an exclusive licence and/or a non-exclusive licence for contributions from authors who are: i) UK Crown employees; ii) where BMJ has agreed a CC-BY licence shall apply, and/or iii) in accordance with the terms applicable for US Federal Government officers or employees acting as part of their official duties; on a worldwide, perpetual, irrevocable, royalty-free basis to BMJ Publishing Group Ltd ("BMJ") its licensees and where the relevant Journal is co-owned by BMJ to the co-owners of the Journal, to publish the Work in this journal and any other BMJ products and to exploit all rights, as set out in our licence.

The Submitting Author accepts and understands that any supply made under these terms is made by BMJ to the Submitting Author unless you are acting as an employee on behalf of your employer or a postgraduate student of an affiliated institution which is paying any applicable article publishing charge ("APC") for Open Access articles. Where the Submitting Author wishes to make the Work available on an Open Access basis (and intends to pay the relevant APC), the terms of reuse of such Open Access shall be governed by a Creative Commons licence – details of these licences and which Creative Commons licence will apply to this Work are set out in our licence referred to above.

Other than as permitted in any relevant BMJ Author's Self Archiving Policies, I confirm this Work has not been accepted for publication elsewhere, is not being considered for publication elsewhere and does not duplicate material already published. I confirm all authors consent to publication of this Work and authorise the granting of this licence.

For peer review only

Computerized Cognitive Training in Parkinson's Disease: A Protocol for a Systematic Review and Updated Meta-Analysis

Hanna Malmberg Gavelin[1, 2], Magdalena Domellöf[2], Isabella Leung[3, 4], Anna Stigsdotter Neely[5], Carsten Finke[6, 7], Amit Lampit[1, 6, 7]*

[1] Academic Unit for Psychiatry of Old Age, University of Melbourne, Parkville, Australia
[2] Department of Psychology, Umeå University, Umeå, Sweden
[3] Healthy Brain Ageing Program, Brain and Mind Centre, University of Sydney, Camperdown, Australia
[4] Central Clinical School, Faculty of Medicine and Health, Charles Perkins Centre, University of Sydney, Camperdown, Australia
[5] Department of Social and Psychological Studies, Karlstad University, Karlstad, Sweden
[6] Department of Neurology, Charité – Universitätsmedizin Berlin, Berlin Germany
[7] Berlin School of Mind and Brain, Humboldt-Universität zu Berlin, Berlin, Germany

* Correspondence concerning this article should be addressed to Amit Lampit, 151 Barry Street, Carlton South VIC 3053, Australia, amit.lampit@unimelb.edu.au

Keywords: Parkinson's disease, cognitive training, systematic review, meta-analysis

Word count: 2011

For peer review only

Abstract

Introduction. Cognitive impairment is recognized as an important non-motor symptom in Parkinson's disease (PD) and there is a need for evidence-based non-pharmacological interventions that may prevent or slow cognitive decline in this patient group. One such intervention is computerized cognitive training (CCT), which has shown efficacious for cognition across older adult populations. This systematic review aims to investigate the efficacy of CCT across cognitive, psychosocial and functional domains for people with PD, and examine study and intervention design factors that could moderate CCT effects on cognition.

Methods and analysis. Randomized controlled trials investigating the effects of CCT in patients with PD without dementia, on cognitive, psychosocial or functional outcomes will be included. The primary outcome is overall cognitive function. Secondary outcomes are domain-specific cognitive function, psychosocial functioning and functional abilities. We systematically searched MEDLINE, Embase and PsycINFO through May 14 2020 to identify relevant literature. Risk of bias will be assessed using the revised Cochrane Risk of Bias tool. Effect sizes will be calculated as standardized mean difference of baseline to post-intervention change (Hedges' *g*) with 95% confidence interval for each eligible outcome measure. Pooling of outcomes across studies will be conducted using random-effects models, accounting for dependency structure of effect sizes within studies. Heterogeneity will be assessed using tau^2 and I^2 statistic. Potential moderators, based on key study and intervention design factors, will be investigated using mixed-effects meta-regression models.

Ethics and dissemination. No ethical approval is required. The findings will be disseminated in a peer-reviewed scientific journal.

PROSPERO registration number: CRD42020185386.

For peer review only

ARTICLE SUMMARY

Strengths and limitations of this study

- Specification of the eligibility criteria for randomized controlled trials of narrowly-defined computerized cognitive training delineates the effects of this intervention from other cognitive intervention approaches in Parkinson's disease.
- Inclusion of a variety of cognitive, psychosocial and functional outcome measures will improve statistical power to inform efficacy across and within domains.
- Accounting for dependency of effect sizes within studies will reduce overestimation of within-study variance and thus underestimation of between-study heterogeneity, especially since individual studies are expected to be underpowered.
- Multiple methods for investigating heterogeneity can inform intervention and study design, but contingent on the number and size of available studies.
- Analyses are limited to group- rather than individual participant data.

For peer review only

INTRODUCTION

Cognitive decline is one of the most common non-motor symptoms in Parkinson's disease (PD).[1] Approximately 20% of people with PD already have mild cognitive impairment (MCI) at diagnosis, with over 40% conversion to dementia 10 years after PD diagnosis, substantially exceeding age-standardized risk.[1] Considering the negative influence of cognitive impairment on quality of life for patients as well as caregivers and the current lack of effective pharmacological treatments,[1] developing interventions that could maintain cognitive function and delay cognitive and functional decline is a critical area for prevention and treatment research in the field.[2]

Cognitive training is a non-pharmacological intervention that has shown efficacious for cognition in older adults across the spectrum from cognitively healthy to dementia.[3] Specifically, computerized cognitive training (CCT) has received widespread attention in recent years as a safe and scalable intervention that can incorporate important intervention design features such as adaptivity of training difficulty and continuous motivational feedback on training performance.[4] In addition to efficacy for cognition, individual trials have reported potential benefits on other symptoms such as mood and freezing of gait.[4]

To date, several systematic reviews and meta-analyses have investigated the efficacy of cognitive training on cognitive function in PD [5-8]. Leung and colleagues[5] identified seven randomized controlled trials (RCTs) and concluded that cognitive training showed modest efficacy for overall cognition in PD, with larger effect sizes observed within individual cognitive domains.[5] Similar results were reported by Lawrence and colleagues,[6] who combined 11 randomized and non-randomized cognitive training trials. More recently, a Cochrane review identified seven RCTs investigating the effects of cognitive training in PD patients with MCI or dementia, reporting imprecise and uncertain evidence for efficacy on global cognition.[7] Approaches for estimating effect sizes across studies varied across reviews,

For peer review only

and none conducted investigations of heterogeneity. Finally, a recent systematic review focusing specifically on CCT reported evidence for cognitive benefits based on seven RCTs; however, no meta-analysis was performed nor were potential effect modifiers explored. [8]

Taken together, previous reviews have shown mixed and inconclusive results and the efficacy of cognitive training in general, and CCT in particular, in people with PD remains uncertain. Furthermore, given the limited number of studies in previous reviews as well as clinical and methodological heterogeneity, the effects of CCT across different cognitive, psychosocial and functional domains as well as design factors that may associated with such effects are still unclear.[4]

Objectives

The aim of this review is to evaluate the efficacy of CCT on cognitive, psychosocial and functional outcomes in persons with PD. Specifically, we aim to:

1. Investigate the efficacy of CCT on cognitive, psychosocial and daily function in PD, in comparison to active or passive control.
2. Examine study and intervention design factors that could moderate CCT effects on cognitive function across studies.
3. Evaluate the strength and quality of the evidence for CCT in PD.
4. Suggest recommendations for future research and practice in the field.

METHODS AND ANALYSIS

This protocol adheres to the Preferred Reporting Items for Systematic Review and Meta-analysis Protocols (PRISMA-P) guidelines[9] and the protocol was prospectively registered with PROSPERO (CRD42020185386). The PRISMA-P checklist can be found in the online

For peer review only

supplementary information. This review updates and further specifies our previous systematic review on cognitive training in PD.[5]

Eligibility criteria

Consistent with our previous systematic reviews of CCT,[10-12] we will include studies that meet the following criteria:

Types of studies

RCTs studying the effects of CCT on one or more cognitive, psychosocial or functional outcome in patients with PD. Eligible studies will provide neuropsychological testing at baseline and post CCT intervention. Randomised crossover trials will be included but only the first treatment phase will be considered and used for analysis. Non-randomised trials will be excluded. Unpublished RCTs or those published as conference abstracts, theses or monographs will be eligible if data needed for analysis and appraisal can be obtained from the authors.

Types of participants

Patients with PD (any age and aetiology), either cognitively healthy, with subjective cognitive decline or MCI. Studies including only or mainly people with dementia will be excluded. Studies reporting the results from a population with mixed populations (e.g., MCI and dementia) will be eligible if the results for the eligible population are reported or can be obtained separately or if the eligible population (e.g., PD with MCI) constitutes ≥50% of the sample.

For peer review only

Types of interventions

Minimum of 4 h of practice on standardized computerized tasks or video games with clear cognitive rationale, administered on personal computers, mobile devices or gaming consoles. Interventions can be delivered individually or in group settings, with or without therapist supervision. Studies combining CCT with other non-pharmacological interventions (e.g., physical exercise, brain stimulation) or with pharmacological interventions will be eligible as long as the CCT condition is the only key difference between the two groups (i.e., study design allows to delineate the effect of CCT from the composite intervention). Studies will not be included if: (1) more than 50% of total intervention time was not CCT; (2) the intervention does not involve interaction with a computer (e.g., passive viewing or recording of responses by an experimenter); (3) the CCT intervention is based on lab-specific rather than off-the-shelf hardware, which makes it less likely to be relevant to clinical practice.

Types of comparators

Eligible control conditions include wait-list, no-contact and active (e.g., sham CCT, recreational activities) control groups. Combined interventions (e.g., pharmacological, physical exercise) will be eligible if provided similarly to both groups. All eligible controls in multi-arm studies will be included.

Types of outcomes

Eligible outcomes are change in performance from baseline to post-intervention in non-trained measures of cognition (global or domain-specific), assessed through standardised neuropsychological tests or close equivalents (e.g., a computer-based version of a common neuropsychological test). Additional outcomes include quality of life (standardised psychological wellbeing and quality of life questionnaires), mood (e.g., clinical depression

For peer review only

rating scales), subjective cognition and daily function (patient or informant reported activities of daily living questionnaires or standardised measures, e.g., timed IADL). Outcomes will be excluded if they were used as (or closely resemble) training tasks or exploratory in nature (i.e., do not resemble common neuropsychological tests). In studies reporting more than one outcome measure per category, all eligible outcome measures will be included. The primary outcome will be overall cognitive performance.[5 10-12] Secondary outcomes are domain-specific cognitive performance, classified according to the CHC-M framework,[13] subjective cognition, psychosocial functioning and daily function. Outcomes from longitudinal follow-ups will be included when available and meta-analytically investigated if appropriate.

Search strategy

We will search MEDLINE, EMBASE and PsycINFO through the OVID interface for eligible articles. As this is an update of our previous systematic review,[5] the search will be limited to entries from 1 January 2015 and records from the updated search will be combined with eligible trials identified through the systematic literature search in the original version of the review. No restrictions on language or type of publication will be applied. The electronic search will be complemented by hand-searching the references of included articles and previous reviews as well as clinical trial registries. The full search strategy is shown in Table 1. A systematic literature search was conducted on May 14 2020.

Table 1

Search strategy

# 1	((cognit* or attention or neurocognit* or neuropsycholog* or memory or mental or reasoning or executive) adj2 (interven* or training* or rehabilitat* or remediat* or stimulat* or activit* or enhanc* or exercis* or retrain*)).mp.
# 2	((brain) adj2 (training* or rehabilitat* or remediat* or retrain*)).mp.
# 3	(speed adj3 training).mp
# 4	(video gam* or videogam* or wii or computer gam* or virtual reality).mp.

For peer review only - http://bmjopen.bmj.com/site/about/guidelines.xhtml

# 5	1 or 2 or 3 or 4
# 6	parkinson$.mp
# 7	exp Parkinson's disease/
# 8	exp Parkinsonism/
# 9	6 or 7 or 8
# 10	5 and 9
# 11	limit 10 to yr="2015 -Current"

Study selection

Literature search results will be uploaded to a single Covidence library. Duplicates will be removed and articles identified from other sources will be added. Initial screening for eligibility based on titles and abstracts will be conducted by two independent reviewers. Full-text screening of potentially relevant articles will be conducted by two independent reviewers and disagreements resolved by consensus or by involvement of a third reviewer.

Data extraction

Data will be extracted to a piloted Excel spreadsheet by one reviewer and a senior reviewer will check the data. Any disagreements will be resolved by consensus or by involvement of a third reviewer if necessary. If any additional information is needed, we will contact the corresponding authors of the studies. The following data items will be extracted:

- Study information: first author, year of publication, study location
- Population: mean age, percent male, mean MMSE score (or equivalent), mean Unified Parkinson's Disease Rating score (or equivalent), disease stage (Hoehn & Yahr Scale or equivalent), disease duration, medication use, cognitive status (normal, subjective cognitive complaints or MCI)
- Intervention: type of CCT, program used, training content, delivery format (supervised or unsupervised), total training duration (hours), session frequency (sessions/week), session length (minutes), total number of sessions, intervention duration (weeks), adjacent treatments

For peer review only - http://bmjopen.bmj.com/site/about/guidelines.xhtml

- Comparator: type of control, control group activity
- Outcome: name of test, summary data for each group (e.g., mean, standard deviation, sample size) at baseline and post intervention, cognitive domain

Intention-to-treat data will be preferred if reported. Data will be extracted as means and standard deviation for each time point if reported. If such information is not available, data in other formats (e.g., mean change and standard deviation) will be used if the article provides sufficient information to reliably calculate standardised mean difference. If these data are unavailable, authors will be contacted to obtain missing data.

Risk of bias assessment

Risk of bias in individual RCTs will be assessed using the revised Cochrane Risk of Bias tool (RoB 2).[14] Low, high or some concerns risk of bias will be determined for each of the following domains:

1. Bias arising from the randomization process
2. Bias due to deviations from intended interventions
3. Bias due to missing outcome data
4. Bias in measurement of the outcome
5. Bias in selection of the reported result
6. Overall bias

Studies with “some concerns” or “high” risk of bias in domains 3 or 4 will be considered as having some concerns or high risk of bias, respectively. Two independent reviewers will assess the risk of bias and disagreements will be resolved by consensus or consulting a third reviewer if necessary.

For peer review only

Data synthesis

Analyses will be conducted using the packages metafor, metaSEM, robumeta and clubSandwich for R. Between-group differences in change from baseline to post-intervention will be converted to standardized mean differences and calculated as Hedges' *g* with 95% confidence interval for each eligible outcome measure. Pooling of outcomes across studies will be conducted using random-effects models, accounting for dependency structure of effect sizes within studies.[15 16] Sensitivity analyses for the primary outcome will be conducted by comparing results from multilevel and robust variance estimation models. Analyses of secondary outcomes will be contingent on the availability of at least three studies for analysis.

Heterogeneity across studies will be quantified using tau^2 and expressed as a proportion of overall observed variance using the I^2 statistic.[17 18] Prediction intervals will be calculated to assess the dispersion of effects across settings.[19] Provided sufficient statistical power for investigations of heterogeneity,[20] potential moderators will be investigated using mixed-effects meta-regression models. The following moderators will be tested, if warranted: training content and type; control content and type; population (clinical or cognitive status); delivery format; training dose and frequency. Meta-regressions will not be conducted if heterogeneity in the overall model is negligible (i.e., $tau^2 < 0.01$) or when there are less than three studies within a planned subgroup. If warranted, potential interactions across moderators will be tested on an exploratory basis using multivariate meta-regression or network meta-analysis.

Meta-bias(es)

Small-study effect will be assessed by visually inspecting funnel plots of effect size vs standard error. [21] If at least 10 studies are available, small study effect will be formally tested using a multivariate analogue of the Egger's test,[22] i.e., a meta-regression using standard error

For peer review only - http://bmjopen.bmj.com/site/about/guidelines.xhtml

as covariate. Subgroup analysis of the primary outcome will be conducted based on overall RoB 2 scores.

Confidence in cumulative evidence

The strength of the evidence will be assessed and summarized qualitatively based on risk of bias for individual studies, precision of the effect estimates, heterogeneity across studies (including prediction intervals) and evidence for small study effects, with additional sensitivity analyses conducted if warranted.

Patient and public involvement

Patients and/or the public will not be involved in this study.

Ethics and dissemination

No formal ethical assessment or informed consent is required for this study. The findings of the study will be summarised in a manuscript which will be submitted for publication in a peer-reviewed scientific journal.

Author Contributions. Guarantor: AL. Design and conceptualisation: HMG and AL. Data collection: HMG, MD and IL. Risk of bias assessment: HMG, MD and IL. Data analysis and interpretation: HMG, MD, IL, ASN, CF and AL. Drafting and revising the manuscript: HMG, MD, IL, ASN, CF and AL.

Funding. This work was supported by a CR Roper Fellowship from the University of Melbourne provided to AL (2020-1), and by the Swedish Research Council (2017-02371) as well as the Swedish Research Council for Health, Working-Life and Welfare (2014-01654) awarded to ASN.

For peer review only

Competing interests. None declared.

For peer review only

References

1. Aarsland D, Creese B, Politis M, et al. Cognitive decline in Parkinson disease. *Nat Rev Neurol* 2017;13(4):217-31. doi: 10.1038/nrneurol.2017.27.
2. Goldman JG, Vernaleo BA, Camicioli R, et al. Cognitive impairment in Parkinson's disease: a report from a multidisciplinary symposium on unmet needs and future directions to maintain cognitive health. *NPJ Parkinsons Dis* 2018;4:19. doi: 10.1038/s41531-018-0055-3.
3. Gavelin HM, Lampit A, Hallock H, et al. Cognition-Oriented Treatments for Older Adults: a Systematic Overview of Systematic Reviews. *Neuropsychol Rev* 2020;30(2):167-93. doi: 10.1007/s11065-020-09434-8.
4. Walton CC, Naismith SL, Lampit A, et al. Cognitive Training in Parkinson's Disease. *Neurorehabil Neural Repair* 2017;31(3):207-16. doi: 10.1177/1545968316680489.
5. Leung IH, Walton CC, Hallock H, et al. Cognitive training in Parkinson disease: A systematic review and meta-analysis. *Neurology* 2015;85(21):1843-51. doi: 10.1212/WNL.0000000000002145.
6. Lawrence BJ, Gasson N, Bucks RS, et al. Cognitive training and noninvasive brain stimulation for cognition in Parkinson's disease: a meta-analysis. *Neurorehabil Neural Repair* 2017;31(7):597-608. doi: 10.1177/1545968317712468.
7. Orgeta V, McDonald KR, Poliakoff E, et al. Cognitive training interventions for dementia and mild cognitive impairment in Parkinson's disease. *Cochrane Database Syst Rev* 2020;2:CD011961. doi: 10.1002/14651858.CD011961.pub2.
8. Nousia A, Martzoukou M, Tsouris Z, et al. The beneficial effects of computer-based cognitive training in Parkinson's Disease: A systematic review. *Arch Clin Neuropsychol* 2020;35(4):434-47.
9. Moher D, Shamseer L, Clarke M, et al. Preferred reporting items for systematic review and meta-analysis protocols (PRISMA-P) 2015 statement. *Systematic Reviews* 2015;4:1. doi: 10.1186/2046-4053-4-1.
10. Lampit A, Hallock H, Valenzuela M. Computerized cognitive training in cognitively healthy older adults: a systematic review and meta-analysis of effect modifiers. *PLoS Med* 2014;11(11):e1001756. doi: 10.1371/journal.pmed.1001756.
11. Hill NT, Mowszowski L, Naismith SL, et al. Computerized Cognitive Training in Older Adults With Mild Cognitive Impairment or Dementia: A Systematic Review and Meta-Analysis. *Am J Psychiatry* 2017;174(4):329-40. doi: 10.1176/appi.ajp.2016.16030360.
12. Lampit A, Heine J, Finke C, et al. Computerized Cognitive Training in Multiple Sclerosis: A Systematic Review and Meta-analysis. *Neurorehabil Neural Repair* 2019;33(9):695-706. doi: 10.1177/1545968319860490.
13. Webb SL, Loh V, Lampit A, et al. Meta-Analysis of the Effects of Computerized Cognitive Training on Executive Functions: a Cross-Disciplinary Taxonomy for Classifying Outcome Cognitive Factors. *Neuropsychol Rev* 2018;28(2):232-50. doi: 10.1007/s11065-018-9374-8.
14. Sterne JA, Savovic J, Page MJ, et al. RoB 2: a revised tool for assessing risk of bias in randomised trials. *BMJ* 2019;366:l4898. doi: 10.1136/bmj.l4898.
15. Cheung MW. A Guide to Conducting a Meta-Analysis with Non-Independent Effect Sizes. *Neuropsychol Rev* 2019;29(4):387-96. doi: 10.1007/s11065-019-09415-6.
16. Hedges LV, Tipton E, Johnson MC. Robust variance estimation in meta-regression with dependent effect size estimates. *Res Synth Methods* 2010;1(1):39-65. doi: 10.1002/jrsm.5.
17. Borenstein M, Higgins JP, Hedges LV, et al. Basics of meta-analysis: I(2) is not an absolute measure of heterogeneity. *Res Synth Methods* 2017;8(1):5-18. doi: 10.1002/jrsm.1230.
18. Higgins JP, Thompson SG. Quantifying heterogeneity in a meta-analysis. *Stat Med* 2002;21(11):1539-58. doi: 10.1002/sim.1186.
19. Riley RD, Higgins JP, Deeks JJ. Interpretation of random effects meta-analyses. *BMJ* 2011;342:d549. doi: 10.1136/bmj.d549.
20. Hedges LV, Pigott TD. The power of statistical tests for moderators in meta-analysis. *Psychol Methods* 2004;9(4):426-45. doi: 10.1037/1082-989X.9.4.426.

For peer review only

21. Sterne JA, Sutton AJ, Ioannidis JP, et al. Recommendations for examining and interpreting funnel plot asymmetry in meta-analyses of randomised controlled trials. *BMJ* 2011;343:d4002. doi: 10.1136/bmj.d4002.

22. Egger M, Davey Smith G, Schneider M, et al. Bias in meta-analysis detected by a simple, graphical test. *BMJ* 1997;315(7109):629-34.

For peer review only

Reporting checklist for protocol of a systematic review.

Based on the PRISMA-P guidelines.

Instructions to authors

Complete this checklist by entering the page numbers from your manuscript where readers will find each of the items listed below.

Your article may not currently address all the items on the checklist. Please modify your text to include the missing information. If you are certain that an item does not apply, please write "n/a" and provide a short explanation.

Upload your completed checklist as an extra file when you submit to a journal.

In your methods section, say that you used the PRISMA-Preporting guidelines, and cite them as:

Moher D, Shamseer L, Clarke M, Ghersi D, Liberati A, Petticrew M, Shekelle P, Stewart LA. Preferred Reporting Items for Systematic Review and Meta-Analysis Protocols (PRISMA-P) 2015 statement. Syst Rev. 2015;4(1):1.

		Reporting Item	Page Number
**Title**			
Identification	#1a	Identify the report as a protocol of a systematic review	1
Update	#1b	If the protocol is for an update of a previous systematic review, identify as such	1
**Registration**			
	#2	If registered, provide the name of the registry (such as PROSPERO) and registration number	2
**Authors**			
Contact	#3a	Provide name, institutional affiliation, e-mail address of all protocol authors; provide physical mailing address of corresponding author	1

For peer review only

Contribution	#3b	Describe contributions of protocol authors and identify the guarantor of the review	12
**Amendments**			
	#4	If the protocol represents an amendment of a previously completed or published protocol, identify as such and list changes; otherwise, state plan for documenting important protocol amendments	Amendments will be recorded in PROSPERO
**Support**			
Sources	#5a	Indicate sources of financial or other support for the review	12
Sponsor	#5b	Provide name for the review funder and / or sponsor	12
Role of sponsor or funder	#5c	Describe roles of funder(s), sponsor(s), and / or institution(s), if any, in developing the protocol	n/a
**Introduction**			
Rationale	#6	Describe the rationale for the review in the context of what is already known	4-5
Objectives	#7	Provide an explicit statement of the question(s) the review will address with reference to participants, interventions, comparators, and outcomes (PICO)	5
**Methods**			
Eligibility criteria	#8	Specify the study characteristics (such as PICO, study design, setting, time frame) and report characteristics (such as years considered, language, publication status) to be used as criteria for eligibility for the review	6-8
Information sources	#9	Describe all intended information sources (such as electronic databases, contact with study authors, trial registers or other grey literature sources) with planned dates of coverage	8

For peer review only

Search strategy	#10	Present draft of search strategy to be used for at least one electronic database, including planned limits, such that it could be repeated	8-9
Study records - data management	#11a	Describe the mechanism(s) that will be used to manage records and data throughout the review	9
Study records - selection process	#11b	State the process that will be used for selecting studies (such as two independent reviewers) through each phase of the review (that is, screening, eligibility and inclusion in meta-analysis)	9
Study records - data collection process	#11c	Describe planned method of extracting data from reports (such as piloting forms, done independently, in duplicate), any processes for obtaining and confirming data from investigators	9-10
Data items	#12	List and define all variables for which data will be sought (such as PICO items, funding sources), any pre-planned data assumptions and simplifications	9-10
Outcomes and prioritization	#13	List and define all outcomes for which data will be sought, including prioritization of main and additional outcomes, with rationale	7-8
Risk of bias in individual studies	#14	Describe anticipated methods for assessing risk of bias of individual studies, including whether this will be done at the outcome or study level, or both; state how this information will be used in data synthesis	10
Data synthesis	#15a	Describe criteria under which study data will be quantitatively synthesised	11
Data synthesis	#15b	If data are appropriate for quantitative synthesis, describe planned summary measures, methods of handling data and methods of combining data from studies, including any planned exploration of consistency (such as I2, Kendall's τ)	11
Data synthesis	#15c	Describe any proposed additional analyses (such as sensitivity or subgroup analyses, meta-regression)	11

For peer review only

Data synthesis	#15d	If quantitative synthesis is not appropriate, describe the type of summary planned	n/a
Meta-bias(es)	#16	Specify any planned assessment of meta-bias(es) (such as publication bias across studies, selective reporting within studies)	11-12
Confidence in cumulative evidence	#17	Describe how the strength of the body of evidence will be assessed (such as GRADE)	12

The PRISMA-P checklist is distributed under the terms of the Creative Commons Attribution License CC-BY 4.0. This checklist was completed on 15. May 2020 using https://www.goodreports.org/, a tool made by the EQUATOR Network in collaboration with Penelope.ai

For peer review only
